# Sexually Transmitted Infections and Associated Risk Factors Among Male Clients of Sex Workers: A Cross-Sectional Pilot Project in Antwerp, Belgium

**DOI:** 10.3389/frph.2022.837102

**Published:** 2022-03-07

**Authors:** Tom Platteau, Irith De Baetselier, Heleen Van Mieghem, Achilleas Tsoumanis, Kris Keersmaekers, Lieselot Ooms, Vicky Cuylaerts, Eric Florence

**Affiliations:** ^1^Department of Clinical Sciences, Institute of Tropical Medicine, Antwerp, Belgium; ^2^Department of Psychology, Open University, Heerlen, Netherlands; ^3^Violett, Antwerp, Belgium; ^4^Department of Public Health, Institute of Tropical Medicine, Antwerp, Belgium

**Keywords:** communicable diseases, diagnostic screening programs, epidemiology, clients of sex workers, sex work, HIV, sexually transmitted diseases (STDs), sexually transmitted infection (STI)

## Abstract

**Introduction:**

Prevalence of sexually transmitted infections (STIs) is increasing in Belgium in recent years. Clients of sex workers form a key population for acquisition of STIs, due to their sexual relations, with or without a condom, with sex workers. STI testing uptake is low among clients of sex workers, and prevalence of STIs remains to be investigated in Belgium. Therefore, we offered STI-testing to clients of sex workers during outreach sessions in Antwerp.

**Methods:**

Time location sampling (TLS) was used to improve representativeness of the sample during ten test sessions in the red light district, Antwerp in May and September 2019 by using a passive approach. Individuals that were interested to get tested for STIs could enter the study. Participants completed an online survey and samples for STI testing were collected. Testing included HIV, syphilis, *Chlamydia trachomatis* (Ct) and *Neisseria gonorrhoeae* (Ng). Test results were communicated *via* a cell phone message (for negative test results) or by phone (for positive test results).

**Results:**

In total, 154 male clients of sex workers with a median age of 38 participated. A total of eight Ct and one Ng infections were detected. TLS analysis revealed a Ct/Ng prevalence of 8.2%. No new HIV nor syphilis infections were detected. Using univariate analysis, testing positive for STI was associated with younger age and anorectal sex with a sex worker. Using multivariate analysis, an STI-positive test result was associated with being younger, having non-Belgian nationality, and being in a relationship.

**Conclusion:**

Our study found a substantial prevalence of Ct/Ng which highlights the need for sensitization and facilitation of STI testing among clients of sex workers. It is difficult to compare results due to the lack of reference material. Moreover, our relatively small convenience sample limits generalizability of results. However, phone counseling (for positive test results) was accepted, linkage to care was provided, and partner notification was facilitated.

## Introduction

Sex workers are considered to be a key population for HIV and sexually transmitted infections (STI) (1, 2). Due to their ubiquitous dyadic sexual relation with their clients, clients are also potentially at risk for acquiring HIV/STI. Therefore, clients are considered to be a key population for STI acquisition, and may serve as “bridge” population to transmit STIs to the general population *via* their sexual relationships with main and casual sex partners (1, 2).

Some male clients of sex workers visit a sex worker as their sole sexual activity, others have sexual contacts with sex workers complementary to the sexual relations with their steady or casual partners. In 2018, 334 clients of Belgian sex workers participated in an online survey (3). The majority (57.2%) reported to be in a relationship (3). Although a substantial proportion (19%) reported inconsistent condom use during vaginal sex with a sex worker, STI screening rate was very low: 42% never got tested; 15% only took a test after a risky sexual contact, 13% tested less than once per year and only 30% tested at least yearly. Even more alarming is the lack of testing among respondents who request condomless sex: 38% of clients who reported condomless oral, 37% condomless vaginal, and 25% who reported condomless anal sex did not take a test (3). Despite this lack of testing among those at highest risk, 14.3% was diagnosed with an STI in the past (3).

Though clients of sex workers may have an increased risk of HIV/STI acquisition, they are highly understudied. In our literature search, we found local studies on HIV and/or STI among non-clinical groups of clients of sex workers in United Kingdom (4), China (5, 6), Togo (7), Benin (8), and Russia (9), yet not one from Belgium. However, these countries have different regulations what makes comparisons challenging. Findings from The Netherlands, a neighboring country of Belgium with comparable legislation, revealed a prevalence of 10.4% STI-diagnoses among a group of clients of sex workers in a clinical setting (10).

Belgian figures of HIV/STI infection proportions are consistent with the EU/EEA figures. Yearly numbers of HIV diagnoses in Belgium decreased with 17% between 2013 and 2018, which is a smaller decline compared to the EU/EEA region (−22.7%) (11). STI-diagnoses rose dramatically between 2014 and 2018 in Belgium. Estimated mean incidence per 100.000 inhabitants of *Chlamydia trachomatis* (Ct) rose from 50.3 to 77.1 (+53%), for *Neisseria gonorrhoeae* (Ng) from 10.3 to 25.95 (+152%), and for syphilis from 7.4 to 21.58 (+192%; personal communication with National Institute of Public Health, Sciensano) over those years. Increased testing among key populations may account (partly) for this increase.

Results for 2019 from an Antwerp health care institute designated for sex workers (Violett) revealed that 13.6% of tested sex workers were diagnosed with Chlamydia, and 9.9% with Gonorrhea when intensively screened (12). Genital Chlamydia and Gonorrhea infections account for less than half of the infections (45 and 33%, respectively). Pharyngeal and anal STI infections are frequently detected among sex workers: for Gonorrhea, 37% of the diagnosed infections were pharyngeal, and 30% were anal infections; for Chlamydia, 13% and 42% were pharyngeal and anal, respectively (12). Percentages for HIV (0.6%) and syphilis infections (1.6%) among sex workers in Belgium are relatively stable over the years (12).

Consistent with the 90-90-90 goals to achieve HIV elimination (13), the World Health Organization (WHO) sets the ambitious goal to eliminate STIs by 2030 (14). One of their key actions is to understand the STI epidemic and to define the possible key populations in each country. Indeed, whereas STI epidemiology varies hugely between regions and countries, a tailored approach, adapted on local needs, should be prioritized (15).

As clients of sex workers are considered a potential key population for HIV and STI (1, 2), testing them may prevent ongoing HIV/STI transmission. Due to feelings of anxiety (for confidentiality breach) and shame, clients of sex workers may not access the healthcare system. We aimed to fill this gap in clinical practice, whereas clients are underserved for testing. For this purpose, clients were tested in the red light district and received their test results *via* a cell phone message (in case of a negative test result) or phone call (positive test result), and were linked to care.

The main objective of the project was to estimate the prevalence for HIV, syphilis, Ct and Ng, and examine associations between an STI diagnosis and potential risk factors for HIV/STI acquisition.

## Materials and Methods

### Study Setting

We organized a prospective cross-sectional pilot study to estimate the prevalence of STIs among clients of sex workers. Violett Antwerp, an organization providing medical and social support to sex workers, opened their offices for clients during specific timeframes (“test sessions”). Clients of sex workers were enrolled during ten test sessions over the span of 2 weeks (1 week in spring and one in autumn 2019). Each testing week included five test sessions.

Sessions lasted 3 or 4 h and were organized during different moments of the day (afternoon, evening, night). An overview of the test sessions is provided as Annex 1. Offices of Violett Antwerp were only accessible for clients of sex workers during test sessions as this could potentially undermine the privacy and perception of a safe environment for sex workers. Outside the dedicated test sessions, clients were referred to traditional testing structures, such as their general practitioner, or existing designated STI-testing centers in Belgium's major cities.

Test sessions were advertised prior to the actual session using leaflets explaining the study in Dutch and English. Additionally, test sessions in fall 2019 were announced *via* designated websites: Redlights.be, Hookers.nl, and Violett's website and Facebook page. A study booth was placed in the street and a passive approach was used. Using a passive approach, we do not actively reach out to potential participants, but inform them when they visit the booth. In case an individual showed interest in participation, a project collaborator provided additional information and guided him to the Violett offices for participation.

### Study Participants

Having had oral, vaginal or anal sex with a sex worker within the previous 12 months was a prerequisite for participation in the study. Other inclusion criteria were being at least 18 years old, being able to understand Dutch or English, accepting to provide contact details (phone number), and agreeing to fill in a short online survey. Seemingly being under influence of drugs or alcohol at the moment of the test was an exclusion criterion which was subjectively assessed by the project collaborator.

Providing informed consent was mandatory to participate. Due to the sensitive information collected, “informed consent forms” were anonymized by removing names and phone numbers upon publication of the final report (June 30, 2020). Participants were informed that they will not have access to their test results at a later time because any link between individual results and identifiable information is removed. Ethical clearance for this study was obtained *via* the Ethics Committee of the University Hospital Antwerp in Belgium.

Participation to the project was free of charge. No incentives were provided for participation.

### Procedures

At the sexual health center, participants received information on the project. They filled in an online survey, and samples for STI testing were collected.

The electronic self-reported survey was developed using Formsite software. The following demographic and behavioral variables of interest were collected: age, nationality, relationship status, type of sexual acts with sex worker (oral, vaginal, anal sex), frequency of sexual intercourse with sex worker, gender of sex worker (female, male, transgender), condom use during sexual activity, substance use when having sexual intercourse with sex worker (including type of substance), and HIV/STI testing history and diagnoses. The survey is provided as Annex 2.

After completion of the survey, blood *via* venepuncture (HIV and syphilis testing) and first-void urine (Ct/Ng testing) were collected. During the blood sampling procedure, the nurse explicitly asked if the participant has had receptive anal sex with a (male or transgender) sex worker. In this case, an ano-rectal regular flocked swab (Copan Diagnostics, Brescia, Italy) for anal Ct/Ng testing was self-collected by the participant.

Blood and urine samples were kept refrigerated (2–8°C) until transportation to the laboratory. Swabs were kept frozen (−20°C). Samples were brought to the laboratory within 24 h during weekdays; if a test session took place in the weekend, samples were brought to the laboratory on Monday.

### Laboratory Methods

Blood samples were tested for HIV according to a validated HIV screening algorithm which includes two 4th generation HIV serology assays [VIDAS HIV Duo Quick (Biomerieux, France) and Genscreen Ultra HIV Ag-Ab (Bio-Rad, France)] and one confirmation assay Geenius HIV 1/2 Confirmatory Assay (Bio-Rad, France). Syphilis testing included TPA (Vitros 5600, Ortho Clinical Diagnostics, USA) and RPR (Macro-Vue, BD, USA) testing. Molecular Ct/Ng testing on urine and anorectal swabs was performed using the Abbott RealTime CT/NG assay (Abbott Molecular, Des Plaines, IL) according to the manufacturer's instructions. In case a sample was positive for Ng, an in-house confirmation molecular assay was performed (16).

### Result Reporting

In case of a negative test result, results were communicated *via* a short cell phone message (sms) using the Institute of Tropical Medicine's (ITM) account on Twilio, a cell phone messaging service. The phone number appearing on participants' cell phone cannot be linked to either of the partnering organizations, nor the project to safeguard participants' privacy. In order to avoid complications when someone reads the sms, the message was kept as neutrally as possible nor did we mention STIs: “Dear, we want to inform you that all test results are OK. Best regards”. In case of a positive test result, participants were called by a trained counselor to communicate the results and refer them for further follow-up and treatment. Precautions to assure result communication to the right person were taken by asking an individual participant's “code word” at the start of the phone call. Counseling for intake of PrEP (pre-exposure prophylaxis for HIV, if clients meet the criteria) was provided during this phone call.

### Statistical Analysis

A sample size of 100 participants was decided, which would yield a 5% error of margin in the two-sided 95% confidence interval, assuming a prevalence of 7% for any STI among clients of sex workers. A simplified version of the Time Location Sampling (TLS) was used to calculate sampling weights for each participant. TLS has been used successfully in previous studies and has demonstrated to be an effective and reliable method for gathering both behavioral and biological data in “hard-to-reach” populations (17–21).

Sampling weights were the inverse of selection probability, which was the product of attendance frequency of each participant in the last 3 months and the fraction of persons recruited over the estimated total number of potential participants at each session. Continuous characteristics were described in terms of medians and interquartile ranges (IQR) and categorical characteristics in terms of counts and percentages. Prevalence of STIs were calculated using sampling weights and estimations are presented as proportions with 95% confidence intervals (CI). All data analysis was carried out taking the sampling weights into consideration. Association between categorical variables was assessed using Chi-square and Fisher's exact test, with a significance level of 5%. Logistic regression was additionally used to identify risk factors for the presence of STIs in the sample, both in a univariate and multivariate way.

A first approach for multivariate analysis was data-driven. Statistically significant variables from the univariate analysis were included, yet the model was not significant. The second approach was to select predictors based on significance in the univariate analysis at a 10% significance level and on the study group's expertise to be included in a multivariate regression model with “STI diagnosis” as outcome variable. We used stepwise backward elimination for model selection. Analyses were done using R version 3.5.1 (22), and all estimates were calculated using the package survey (23).

### Ethical Statement

This study was approved by the Institutional Review Board (IRB) of the Institute of Tropical Medicine (Antwerp, Belgium; ref. 1288/19) and the Ethics Committee of the University Hospital Antwerp (Antwerp, Belgium; ref. 19/13/170). During the process of approval all conditions, procedures and rights of participants are examined. Only if the research meets all criteria, approval is granted.

## Results

### Descriptive Results

During ten test sessions, 154 men participated. Median age was 38 years old (IQR 28–50). Median number of visits to sex workers per month was one, or three in the past 3 months (IQR 2–6). Participants originated from 19 different countries. An overview of demographic variables is provided in Table 1.

**Table 1:** Overview of demographic information of participants.

**Variable**	**Number (%)**
Relationship status	
Single	88 (57.1)
In a relationship with a female partner	65 (42.2)
In a relationship with a male partner	1 (0.7)
Country of origin	
Belgium	95 (61.7)
The Netherlands	25 (16.2)
Afghanistan	5 (3.3)
India	4 (2.6)
Lithuania	4 (2.6)
Originating from another country	21 (13.6)
Gender of sex worker^*^	
Female	153 (99.4)
Male	5 (3.2)
Transgender	8 (5.2)
Sexual activities with sex worker^*^	
Oral sex	110 (71.4)
Vaginal sex	146 (94.8)
Anal sex	23 (14.9)

* *For this question multiple answers were possible*.

Of 110 men (71.4%) who reported oral sex, 71 (64.5%) reported inconsistent condom use. Of participants reporting vaginal sex (*n* = 146), 41 (28.1%) reported inconsistent condom use. During anal sex, inconsistent condom use was reported by 60.9% of respondents (*n* = 14/23).

Substance use during sexual intercourse with a sex worker was reported by 29 respondents (18.8%). Most common reported substances were cannabis (*n* = 19; 65.5%), alcohol (*n* = 16; 55.2%), poppers (*n* = 9; 31.0%), cocaine (*n* = 4; 13.8%) and MDMA (*n* = 3; 10.3%).

About half of participants (*n* = 83; 53.9%) had been tested for STIs at least once. A description of testing history, frequency of testing, and self-reported STI diagnoses in the past is provided in Table 2.

**Table 2:** Testing history, and diagnosed STIs in the past.

**Variable**	**Number (%)**
Were you tested before?	
No	71 (46.1)
Yes, after a risky contact	24 (15.6)
Yes, less than once yearly	26 (16.9)
Yes, at least once yearly	33 (21.4)
Where were you tested?	
GP/family physician	35 (66.3)
HIV/STI clinic	13 (15.7)
Other physician	5 (6.0)
Other	10 (12.0)
Have you been diagnosed with an STI in the past?	
No	85 (55.2)
Yes	23 (14.9)
I don't know	46 (29.9)

*STI, Sexually Transmitted Infections; GP, general practitioner*.

### STI Test Results

One man (0.66%; 95% CI: 0.035–3%) was HIV positive but previously aware of his infection. He tested negative for the other STIs and is therefore included as STI negative in this analysis. Eight participants tested positive for Chlamydia, of whom one also tested positive for Gonorrhea. Using TLS analysis, point prevalence is 8.2% (95% CI: 3.1–17.0) for Chlamydia and/or Gonorrhea. No syphilis infections were found.

### Univariate Analysis

Relevant variables of participants with and without STI-diagnosis in the study were compared. Participants with a positive STI test result were younger compared to STI-negative participants (mean age = 31.1 vs. 40.2; *p* = 0.01). An overview of the proportions, and *p*-values for the other selected variables is provided in Table 3.

**Table 3:** Univariate analysis of relevant variables with STI-test result as dependent variable.

**Variable**	**STI-negative (*n* = 146) *N* (%)**	**STI-positive (*n* = 8) *N* (%)**	***p*-value**
**Country of origin**			0.483
Belgium	91 (62.3)	4 (50.0)	
Other	55 (37.7)	4 (50.0)	
**Relationship status**			0.289
Single	85 (58.2)	3 (37.5)	
In a relation	61 (41.8)	5 (62.5)	
**Gender of the sex worker**			
*Female*			1
Yes	145 (99.3)	8 (100.0)	
No	1 (0.7)	0 (0.0)	
*Male*			1
Yes	5 (3.4)	0 (0.0)	
No	141 (96.6)	8 (100.0)	
*Transgender*			0.057
Yes	6 (4.1)	2 (25.0)	
No	140 (95.9)	6 (75.0)	
**Sexual activities**			
*Oral sex with sex worker*			0.441
Yes	103 (70.5)	7 (87.5)	
No	43 (29.5)	1 (12.5)	
*Vaginal sex with sex worker*			0.354
Yes	139 (95.2)	7 (87.5)	
No	7 (4.8)	1 (12.5)	
*Anal sex with sex worker*			0.018
Yes	19 (13.0)	4 (50.0)	
No	127 (87.0)	4 (50.0)	
**Condom use**			
*During oral sex*			0.679
Consistent condom use	36 (35.0)	3 (42.9)	
Inconsistent condom use	67 (65.0)	4 (57.1)	
*During vaginal sex*			0.401
Consistent condom use	101 (72.7)	4 (57.1)	
Inconsistent condom use	38 (27.3)	3 (42.9)	
*During anal sex*			1
Consistent condom use	8 (42.1)	1 (25.0)	
Inconsistent condom use	11 (57.9)	3 (75.0)	
**Risk factors and testing history**			
*Substance use*			0.173
Yes	26 (17.8)	3 (37.5)	
No	120 (82.2)	5 (62.5)	
*Ever tested for STI*			0.472
Yes	80 (54.8)	3 (37.5)	
No	66 (45.2)	5 (62.5)	
*Ever STI diagnosis*			1
Yes	22 (15.1)	1 (12.5)	
No	80 (54.8)	5 (62.5)	
I don't know	44 (30.1)	2 (25.0)	

*STI, Sexually Transmitted Infections*.

### Multivariate Analysis

As described in Section Material and Methods, the data-driven approach did not provide any significant prediction model. By using a combined data- and content-approach, “having an STI diagnosis” was predicted by non-Belgian nationality, younger age and being in a relationship, as presented in Table 4.

**Table 4:** Multivariate analysis with STI-test result as dependent variable.

**Variable**	**Odds ratio**	**95% confidence interval**	***P*-value**
Non-Belgian nationality	4.24	0.81–22.24	0.09
Age	0.91	0.86– 0.97	0.006
Being in a relationship	7.84	1.42–43.37	0.02
Frequency of visiting a sex worker			ns
Condom use during oral sex			ns
Condom use during vaginal sex			ns
Condom use during anal sex			ns
Drug use			ns

*ns, not significant*.

The model described is the best fit for a positive STI-test in the group of participants. Even though the coefficient for the variable “Non-Belgian nationality” (*p* = 0.09) is not significant, the variable is significant for the model.

## Discussion

The primary outcome of the project, testing a group of clients and thereby filling an existing gap, was successfully implemented. The aim was to reach 100 participants during ten test sessions, and we achieved to test 154.

Point prevalence of STIs was substantial: 8.2%. The most often diagnosed STI was Chlamydia, and one participant tested positive for Chlamydia and Gonorrhea. One participant was previously aware of his HIV-positive status, and in follow-up in an HIV treatment center. He reported sexual intercourse with male and female sex workers. During oral and vaginal sex, he reported consistent condom use. He reported no anal sex with a sex worker.

It remains difficult to compare STI prevalence among clients of sex workers in a non-clinical setting in Belgium and Europe. Therefore, these results should be confirmed in other countries, regions, and cities where collaboration between HIV/STI testing centers, laboratories and sexual health organizations for sex workers is feasible. This collaboration seems a prerequisite to set up a successful project for STI testing among clients of sex workers.

Interesting characteristics to assess among clients of sex workers in future projects include some behavioral aspects, perceptions of their sex life with sex workers, reasons and motivations of not having been tested in the past, and potential perception on STI-prevention. The latter could be framed within a broader campaign for sensitization and prevention.

Being younger was significantly associated with an STI diagnosis in the univariate analysis, the latter being consistent with findings in The Netherlands (10). Anal sex with a sex worker was the second aspect that differed between participants with and without STI. This could be explained by the fact that anal Chlamydia and Gonorrhea infection seems to be prevalent among sex workers (12, 24).

A model to assess vulnerability by predicting STI diagnosis was developed. Being non-Belgian, younger and in a relationship showed increased vulnerability for an STI diagnosis. It is early to draw conclusions from these findings, but we hypothesize that the hidden aspect of visiting sex workers (relationship status) and access to health services (nationality) may impact health seeking behavior of clients. Surprisingly, anal sex with a sex worker did not lead to a significant explanation of STI diagnoses *via* logistical regression.

We faced some limitations within the study. The number of participants, as well as the number of STIs, was relatively small. The study population consisted of a convenience sample of clients of sex workers, limiting the generalizability of study results. We did not gather any information of the number of sexual acts in the previous 6 months nor did we gather any information about their sexual partners besides that they had sexual intercourse with a sex worker in the past year. As such, we cannot retrace of whom they acquired the STI. Using a cross-sectional research design, we cannot distinguish causes and consequences. The direction of associations should therefore be treated cautiously. In addition, although Human Papilloma Virus and Herpes Simplex Virus type II are one of the most common STIs, we did not include them in our analysis. Moreover, we did not perform any clinical examination (for genital/anorectal warts or ulcers for example) nor did we ask the patients if they had any symptoms. As such, we can only assume that the STIs detected in this study were asymptomatic. Lastly, the sensitive nature of the collected information and accompanying feelings of shame of participants may have withheld clients from participation.

During the set-up of the project, we searched for a trade-off between safeguarding participants' privacy and offering low-threshold STI testing by obtaining ethical clearance by an Ethics Committee, and discussing each step in the process with ITM's data protection officer (DPO). We respected participants' privacy by deleting all identifying data that were collected during the project.

Communication of the test results by a cell phone message (in case of negative test results) or a phone call (for positive test results) was selected in close collaboration with the DPO. Alternative test results communication channels (email, WhatsApp, etc.) did not safeguard sufficient coping with potential privacy issues and data safety.

Positive results were communicated by phone, and participants were explicitly informed about which STI result was diagnosed to avoid re-testing with their general practitioner or other physician. This is redundant, and expensive. Participants agreed to be contacted *via* telephone, and were aware that (when a test is positive) they should visit a physician for prompt treatment. Counseling by phone offered the possibility to inform and discuss possible prevention measures (PrEP for HIV), treatment for STIs and to emphasize the importance of partner notification.

To conclude, we showed that implementation of an HIV testing approach for clients of sex workers is feasible; clients are motivated if their privacy is sufficiently protected. The prevalence of STIs was high and condom use suboptimal. Therefore, clients need to be sensitized to increase condom use in sexual contacts with sex workers. Moreover, normalizing regular STI testing is critical. This includes sensitizing clients about the importance of testing, improving access to testing, and facilitating low-threshold STI/HIV testing.

## Data Availability Statement

The raw data supporting the conclusions of this article will be made available by the authors, on reasonable request.

## Ethics Statement

The studies involving human participants were reviewed and approved by Institutional Review Board (IRB) of the Institute of Tropical Medicine (Antwerp, Belgium; ref. 1288/19) and the Ethics Committee of the University Hospital Antwerp (Antwerp, Belgium; ref. 19/13/170). The participants provided their written informed consent to participate in this study.

## Author Contributions

TP, ID, HV, AT, KK, and EF: planning. LO, VC, HV, KK, TP, ID, and EF: conduct. TP, ID, AT (statistical analysis), HV, KK, LO, VC, and EF: reporting. TP and ID: writing the first draft of the manuscript. All authors reviewed and approved the manuscript.

## Funding

Funding was received from Gilead Sciences. They however were not involved in the conduct of the study.

## Conflict of Interest

HV and KK were employed by Violett. The remaining authors declare that the research was conducted in the absence of any commercial or financial relationships that could be construed as a potential conflict of interest.

## Publisher's Note

All claims expressed in this article are solely those of the authors and do not necessarily represent those of their affiliated organizations, or those of the publisher, the editors and the reviewers. Any product that may be evaluated in this article, or claim that may be made by its manufacturer, is not guaranteed or endorsed by the publisher.
