# Supplementary material for: Sexually Transmitted Infections and Associated Risk Factors Among Male Clients of Sex Workers: A Cross-Sectional Pilot Project in Antwerp, Belgium
Source: Front Reprod Health. 2022 Mar 7;4:837102. doi: 10.3389/frph.2022.837102 (PMC9580811; doi:10.3389/frph.2022.837102)
Supplement: Supplementary file 1 [file Table_1.pdf]

Supplement 1: Overview of test sessions

**Spring sessions (2019)**	**Session hours**
Tuesday May 14	2 – 5 pm
Wednesday May 15	8 – 12 pm
Thursday May 16	2 – 5 pm
Thursday May 16	8 – 12 pm
Friday May 17	8 – 12 pm
**Autumn sessions (2019)**	
Tuesday September 24	2 – 5 pm
Thursday September 26	8 – 12 pm
Friday September 27	2 – 5 pm
Friday September 27	8 – 12 pm
Saturday September 28	8 – 12 pm
