# Supplementary material for: Sexually Transmitted Infections and Associated Risk Factors Among Male Clients of Sex Workers: A Cross-Sectional Pilot Project in Antwerp, Belgium
Source: Front Reprod Health. 2022 Mar 7;4:837102. doi: 10.3389/frph.2022.837102 (PMC9580811; doi:10.3389/frph.2022.837102)
Supplement: Supplementary file 2 [file Data_Sheet_1.pdf]

Supplement 2: Survey to clients of prostitutes

Screening for participation

i. Did you have sexual intercourse with a prostitute in the past year?
   - No → Thank you for your interest; no participation in the project
   - Yes (go to ii.)

ii. Which sexual acts did you have during your sexual intercourse with a prostitute (multiple answers possible)
   - Oral sex → go to Question 1
   - Vaginal sex → go to Question 2
   - Anal sex → go to Question 3
   - Other → Thank you for your interest; no participation in the project

Personal information

1. What is you day of birth?
   - ...... / …… / ………… (DD/MM/YYYY)

2. Nationality?
   - Belgian
   - Other: ...

3. Are you in a relationship?
   - Yes, with a woman
   - Yes, with a man
   - No

4. With whom do you have sexual contact? (Multiple answers possible)
   - Men
   - Women
   - Transgenders

5. How many times did you pay for sexual services in the past three months?
   - …………..
   -

Sexual intercourse with a prostitute

6. Do you sometimes use a condom when having sex with a prostitute? *(Filter: only show sexual acts that were ticked in screening question ii.)*
   - Oral sex (always, mostly yes, mostly no, never)
   - Vaginal sex (always, mostly yes, mostly no, never)
   - Anal sex (always, mostly yes, mostly no, never)

7. Do you experience other sexual acts with a prostitute?
   - No
   - Yes, specify: ...

Substance use

8. Do you use substances or drugs during sexual intercourse?
    - Yes
    - No

9. If yes, what do you use? (multiple answers possible)
    - Alcohol
    - Cannabis
    - GHB/ GBL
    - MDMA/ XTC
    - Cocaine
    - Speed
    - Poppers
    - Other, specify: ...

Sexually transmitted infections

10. Some people take a general check-up on a regular basis (with their GP, another physician or on the job). Do you, apart from these check-ups, take a specific STI check?
    - Yes, at least once per year
    - Yes, less than once per year
    - Yes, after having had a risky sexual risk (broken condom, sex without condom, experienced symptoms)
    - No, because;
      I am not at risk
      I don't know where I can take a test
      I am anxious for the result
      Other, specify: ...

11. Where do you take a test?
    - GP (family physician)
    - Other physician than GP
    - HIV / STI clinic
    - Self test
    - Other: ...

12. Did you ever acquire an STI?
    - Yes
    - No

13. If yes, which one?
    - Chlamydia
    - Gonorrhea
    - Syphilis
    - Hepatitis A
    - Hepatitis B
    - Hepatitis C
    - HIV
    - Other

Extra

14. If we want to establish this service, are you be willing to disclose your identity (so that we can recuperate the costs via your mutuality)?
    - Yes
    - No
